# Supplementary material for: In Silico Study of piRNA Interactions with the SARS-CoV-2 Genome
Source: Int J Mol Sci. 2022 Aug 31;23(17):9919. doi: 10.3390/ijms23179919 (PMC9456458; doi:10.3390/ijms23179919)
Supplement: Supplementary file 1 [file ijms-23-09919-s001.zip › Figure S1.pdf]

Figure S1. The schemes of spiRNA interaction with full complementarity in clusters of BSs in CDS gRNA SARS-CoV-2 from 12077 nt to 12107 nt

spiRNA; BS, nt; Region; ΔG, kJ/mol; $\Delta G/\Delta G_m$, %; piRNA length, nt

spiR-180819; 12080; CDS; -125; 100; 24
```
5'-GCAACCUUACAAGCUAUAGCCUCA-3'
   ||||||||||||||||||||||||
3'-CGUUGGAAUGUUCGAUAUCGGAGU-5'
```
spiR-184604; 12080; CDS; -125; 100;24
```
5'-GCAACCUUACAAGCUAUAGCCUCA-3'
   ||||||||||||||||||||||||
3'-CGUUGGAAUGUUCGAUAUCGGAGU-5'
```
spiR-187101; 2079; CDS; -132; 100; 25
```
5'-GGCAACCUUACAAGCUAUAGCCUCA-3'
   |||||||||||||||||||||||||
3'-CCGUUGGAAUGUUCGAUAUCGGAGU-5'
```
spiR-188123; 12078; CDS; -138; 100; 26
```
5'-GGGCAACCUUACAAGCUAUAGCCUCA-3'
   ||||||||||||||||||||||||||
3'-CCCGUUGGAAUGUUCGAUAUCGGAGU-5'
```
spiR-188808; 12078; CDS; -138; 100; 26
```
5'-GGGCAACCUUACAAGCUAUAGCCUCA-3'
   ||||||||||||||||||||||||||
3'-CCCGUUGGAAUGUUCGAUAUCGGAGU-5'
```
spiR-188962; 12078; CDS; -138; 100; 26
```
5'-GGGCAACCUUACAAGCUAUAGCCUCA-3'
   ||||||||||||||||||||||||||
3'-CCCGUUGGAAUGUUCGAUAUCGGAGU-5'
```
spiR-189493; 12079; CDS; -132; 100; 25
```
5'-GGCAACCUUACAAGCUAUAGCCUCA-3'
   |||||||||||||||||||||||||
3'-CCGUUGGAAUGUUCGAUAUCGGAGU-5'
```
spiR-189542; 12078; CDS; -138; 100; 26
```
5'-GGGCAACCUUACAAGCUAUAGCCUCA-3'
   ||||||||||||||||||||||||||
3'-CCCGUUGGAAUGUUCGAUAUCGGAGU-5'
```
spiR-189637; 12078; CDS; -138; 100; 26
```
5'-GGGCAACCUUACAAGCUAUAGCCUCA-3'
   ||||||||||||||||||||||||||
3'-CCCGUUGGAAUGUUCGAUAUCGGAGU-5'
```
spiR-190555; 12078; CDS; -138; 100; 26
```
5'-GGGCAACCUUACAAGCUAUAGCCUCA-3'
   ||||||||||||||||||||||||||
3'-CCCGUUGGAAUGUUCGAUAUCGGAGU-5'
```
spiR-190706; 12078; CDS; -138; 100; 26
```
5'-GGGCAACCUUACAAGCUAUAGCCUCA-3'
   ||||||||||||||||||||||||||
3'-CCCGUUGGAAUGUUCGAUAUCGGAGU-5'
```
spiR-190719; 12079; CDS; -132; 100; 25
```
5'-GGCAACCUUACAAGCUAUAGCCUCA-3'
   |||||||||||||||||||||||||
3'-CCGUUGGAAUGUUCGAUAUCGGAGU-5'
```
spiR-190772; 12078; CDS; -138; 100; 26
```
5'-GGGCAACCUUACAAGCUAUAGCCUCA-3'
   ||||||||||||||||||||||||||
3'-CCCGUUGGAAUGUUCGAUAUCGGAGU-5'
```

spiR-191124; 12079; CDS; -132; 100; 25
```
5'-GGCAACCUUACAAGCUAUAGCCUCA-3'
   |||||||||||||||||||||||||
3'-CCGUUGGAAUGUUCGAUAUCGGAGU-5'
```
spiR-191185; 12079; CDS; -132; 100; 25
```
5'-GGCAACCUUACAAGCUAUAGCCUCA-3'
   |||||||||||||||||||||||||
3'-CCGUUGGAAUGUUCGAUAUCGGAGU-5'
```
spiR-191492; 12079; CDS; -132; 100; 25
```
5'-GGCAACCUUACAAGCUAUAGCCUCA-3'
   |||||||||||||||||||||||||
3'-CCGUUGGAAUGUUCGAUAUCGGAGU-5'
```
spiR-192078; 12079; CDS; -132; 100; 25
```
5'-GGCAACCUUACAAGCUAUAGCCUCA-3'
   |||||||||||||||||||||||||
3'-CCGUUGGAAUGUUCGAUAUCGGAGU-5'
```
spiR-192160; 12078; CDS; -138; 100; 26
```
5'-GGGCAACCUUACAAGCUAUAGCCUCA-3'
   ||||||||||||||||||||||||||
3'-CCCGUUGGAAUGUUCGAUAUCGGAGU-5'
```
spiR-194007; 12079; CDS; -132; 100; 25
```
5'-GGCAACCUUACAAGCUAUAGCCUCA-3'
   |||||||||||||||||||||||||
3'-CCGUUGGAAUGUUCGAUAUCGGAGU-5'
```
spiR-194135; 12078; CDS; -138; 100; 26
```
5'-GGGCAACCUUACAAGCUAUAGCCUCA-3'
   ||||||||||||||||||||||||||
3'-CCCGUUGGAAUGUUCGAUAUCGGAGU-5'
```
spiR-194172; 12079; CDS; -132; 100; 25
```
5'-GGCAACCUUACAAGCUAUAGCCUCA-3'
   |||||||||||||||||||||||||
3'-CCGUUGGAAUGUUCGAUAUCGGAGU-5'
```
spiR-194397; 12078; CDS; -138; 100; 26
```
5'-GGGCAACCUUACAAGCUAUAGCCUCA-3'
   ||||||||||||||||||||||||||
3'-CCCGUUGGAAUGUUCGAUAUCGGAGU-5'
```
spiR-194781; 12079; CDS; -132; 100; 25
```
5'-GGCAACCUUACAAGCUAUAGCCUCA-3'
   |||||||||||||||||||||||||
3'-CCGUUGGAAUGUUCGAUAUCGGAGU-5'
```
spiR-345961; 12084; CDS; -125; 100; 24
```
5'-CCUUACAAGCUAUAGCCUCAGAGU-3'
   ||||||||||||||||||||||||
3'-GGAAUGUUCGAUAUCGGAGUCUCA-5'
```
spiR-360432; 12082; CDS; -130; 100; 25
```
5'-AACCUUACAAGCUAUAGCCUCAGAG-3'
   |||||||||||||||||||||||||
3'-UUGGAAUGUUCGAUAUCGGAGUCUC-5'
```
spiR-392668; 12078; CDS; -138; 100; 26
```
5'-GGGCAACCUUACAAGCUAUAGCCUCA-3'
   ||||||||||||||||||||||||||
3'-CCCGUUGGAAUGUUCGAUAUCGGAGU-5'
```
spiR-396601; 12081; CDS; -140; 100; 27
```
5'-CAACCUUACAAGCUAUAGCCUCAGAGU-3'
   |||||||||||||||||||||||||||
3'-GUUGGAAUGUUCGAUAUCGGAGUCUCA-5'
```

spiR-401969; 12078; CDS; -138; 100; 26
```
5'-GGGCAACCUUACAAGCUAUAGCCUCA-3'
   ||||||||||||||||||||||||||
3'-CCCGUUGGAAUGUUCGAUAUCGGAGU-5'
```
spiR-403725; 12079; CDS; -127; 100; 24
```
5'-GGCAACCUUACAAGCUAUAGCCUC-3'
   ||||||||||||||||||||||||
3'-CCGUUGGAAUGUUCGAUAUCGGAG-5'
```
spiR-403862; 12078; CDS; -134; 100; 25
```
5'-GGGCAACCUUACAAGCUAUAGCCUC-3'
   |||||||||||||||||||||||||
3'-CCCGUUGGAAUGUUCGAUAUCGGAG-5'
```
spiR-404008; 12079; CDS; -132; 100; 25
```
5'-GGCAACCUUACAAGCUAUAGCCUCA-3'
   |||||||||||||||||||||||||
3'-CCGUUGGAAUGUUCGAUAUCGGAGU-5'
```
spiR-404056; 12077; CDS; -142; 100; 27
```
5'-AGGGCAACCUUACAAGCUAUAGCCUCA-3'
   |||||||||||||||||||||||||||
3'-UCCCGUUGGAAUGUUCGAUAUCGGAGU-5'
```
spiR-405339; 12079; CDS; -132; 100; 25
```
5'-GGCAACCUUACAAGCUAUAGCCUCA-3'
   |||||||||||||||||||||||||
3'-CCGUUGGAAUGUUCGAUAUCGGAGU-5'
```
spiR-405683; 12079; CDS; -127; 100; 24
```
5'-GGCAACCUUACAAGCUAUAGCCUC-3'
   ||||||||||||||||||||||||
3'-CCGUUGGAAUGUUCGAUAUCGGAG-5'
```
spiR-406209; 12078; CDS; -138; 100; 26
```
5'-GGGCAACCUUACAAGCUAUAGCCUCA-3'
   ||||||||||||||||||||||||||
3'-CCCGUUGGAAUGUUCGAUAUCGGAGU-5'
```
spiR-406508; 12078; CDS; -127; 100; 24
```
5'-GGGCAACCUUACAAGCUAUAGCCU-3'
   ||||||||||||||||||||||||
3'-CCCGUUGGAAUGUUCGAUAUCGGA-5'
```
spiR-406684; 12079; CDS; -132; 100; 25
```
5'-GGCAACCUUACAAGCUAUAGCCUCA-3'
   |||||||||||||||||||||||||
3'-CCGUUGGAAUGUUCGAUAUCGGAGU-5'
```
spiR-410103; 12078; CDS; -138; 100; 26
```
5'-GGGCAACCUUACAAGCUAUAGCCUCA-3'
   ||||||||||||||||||||||||||
3'-CCCGUUGGAAUGUUCGAUAUCGGAGU-5'
```
spiR-1177268; 12082; CDS; -130; 100; 25
```
5'-AACCUUACAAGCUAUAGCCUCAGAG-3'
   |||||||||||||||||||||||||
3'-UUGGAAUGUUCGAUAUCGGAGUCUC-5'
```
