# Supplementary material for: In Silico Study of piRNA Interactions with the SARS-CoV-2 Genome
Source: Int J Mol Sci. 2022 Aug 31;23(17):9919. doi: 10.3390/ijms23179919 (PMC9456458; doi:10.3390/ijms23179919)
Supplement: Supplementary file 1 [file ijms-23-09919-s001.zip › Figure S2+.pdf]

Figure S2. The schemes of 12 piRNAs and spiRNAs interaction with CDS gRNA SARS-CoV-2 from 4670 nt to 29475 nt

<u>piRNA or spiRNA; BS, nt; Region; ΔG, kJ/mol; ΔG/ΔGm, %; piRNA length, nt</u>

piR-2047904; 4670; CDS; -136; 81; 32
```
5'-CUCAAAGUGCCAGCUACAGUUUCUGUUUCUUC-3'
   | |||||| ||||| |||| ||||||||||||
3'-AUGCCUCAAGGUCGUUGUCUAAGACGAAGAAG-5'
```
spiR-2047904; 4670; CDS; -168; 100; 32
```
5'-CUCAAAGUGCCAGCUACAGUUUCUGUUUCUUC-3'
   ||||||||||||||||||||||||||||||||
3'-GAGUUUCACGGUCGAUGUCAAAGACAAAGAAG-5'
```

piR-912075; 9102; CDS; -140; 80; 33
```
5'-AAAGUUUACGCCCUGACACACGUUAUGUGCUCA-3'
   || |||||| |||||||||||| || |||||||
3'-UUACAAGUGAGGGACCGUGUAUCAUCCACGAGU-5'
```
spiR-912075; 9102; CDS; -175; 100; 33
```
5'-AAAGUUUACGCCCUGACACACGUUAUGUGCUCA-3'
   |||||||||||||||||||||||||||||||||
3'-UUUCAAAUGCGGGACUGUGUGCAAUACACGAGU-5'
```

piR-2352720, 9123; CDS; -138; 81; 34
```
5'-GUUAUGUGCUCAUGGAUGGCUCUAUUAUUCAAUU-3'
   |||| ||||||||||||||  |||||||||||||
3'-CGAUCCACGAGCAUUUACCCUGAUAAUAAAUUAA-5'
```
spiR-2352720, 9123; CDS; -170; 100; 34
```
5'-GUUAUGUGCUCAUGGAUGGCUCUAUUAUUCAAUU-3'
   ||||||||||||||||||||||||||||||||||
3'-CAAUACACGAGUACUUACCGAGAUAAUAAGUUAA-5'
```

piR-2490582; 10012; CDS; -142; 83; 33
```
5'-UUACCAACCACCACAAACCUCUAUCACCUCAGC-3'
   || ||||||||||| |||||| ||| |||||||
3'-AAAGGCUGGUGGUGGUUGAAGUUAAAGGAAUCG-5'
```
spiR-2490582; 10012; CDS; -171; 100; 33
```
5'-UUACCAACCACCACAAACCUCUAUCACCUCAGC-3'
   |||||||||||||||||||||||||||||||||
3'-AAUGGUUGGUGGUGUUUGGAGAUAGUGGAGUCG-5'
```

piR-1491787; 17115; CDS; -138; 80; 32
```
5'-UGGCCUAGCUCUCUACUACCCUUCUGCUCGCA-3'
   ||||||||||| ||||| ||| ||| || |||
3'-ACUGGAUCGAGUGACGAAGGGUAGAGGACUGU-5'
```
spiR-1491787; 17115; CDS; -173; 100; 32
```
5'-UGGCCUAGCUCUCUACUACCCUUCUGCUCGCA-3'
   ||||||||||||||||||||||||||||||||
3'-ACCGGAUCGAGAGAUGAUGGGAAGACGAGCGU-5'
```

piR-3555322; 21712; CDS; -136; 82; 34
```
5'-AACUCAGGACUUGUUCUUACCUUUCUUUUCCAAU-3'
   |||||||||||| ||||||||||||| |||||||
3'-UUGAGUUUUGAAAGAGAGUAGAAAGGUAAAGUUA-5'
```
spiR-3555322; 21712; CDS; -166; 100; 34
```
5'-AACUCAGGACUUGUUCUUACCUUUCUUUUCCAAU-3'
   ||||||||||||||||||||||||||||||||||
3'-UUGAGUCCUGAACAAGAAUGGAAAGAAAAGGUUA-5'
```

```
piR-703629; 25672; CDS; -142; 82; 31
5'-CUUUUGCUCGUUGCUGCUGGCCUUGAAGCCC-3'
    ||||||||||||||||| |||||| |||||
3'-CAAAACAAGCGACGACGGACGGGACGUCGGG-5'
spiR-703629; 25672; CDS; -173; 100; 31
5'-CUUUUGCUCGUUGCUGCUGGCCUUGAAGCCC-3'
   |||||||||||||||||||||||||||||||
3'-GAAAACGAGCAACGACGACCGGAACUUCGGG-5'

piR-1525356; 26106; CDS; -136; 80; 32
5'-UGAGCCUGAAGAACAUGUCCAAAUUCACACAA-3'
    ||||||||| |||| ||||||| ||||||||
3'-CCUUGGACUUACUGUCCAGGUUUCAGUGUGUC-5'
spiR-1525356; 26106; CDS; -170; 100; 32
5'-UGAGCCUGAAGAACAUGUCCAAAUUCACACAA-3'
   ||||||||||||||||||||||||||||||||
3'-ACUCGGACUUCUUGUACAGGUUUAAGUGUGUU-5'

piR-2599982; 27070; CDS; -144; 82; 34
5'-CUUCGCAGCGUGUAGCAGGUGACUCAGGUUUUGC-3'
   |||| | | |||||| ||||||||||||||||||
3'-GAAGAAAUUCACGUCUUCCACCAAGUCCAAAACG-5'
spiR-2599982; 27070; CDS; -176; 100; 34
5'-CUUCGCAGCGUGUAGCAGGUGACUCAGGUUUUGC-3'
   ||||||||||||||||||||||||||||||||||
3'-GAAGCGUCGCACAUCGUCCACUGAGUCCAAAACG-5'

piR-806264; 28427; CDS; -142; 83; 33
5'-UGGUUCACCGCUCUCACUCAACAUGGCAAGGAA-3'
   ||| ||||||||||||||||||||||||  |||
3'-ACCCAGUGGUGAGAAUGAGUUGUAUCAUGACCU-5'
spiR-806264; 28427; CDS; -171; 100; 33
5'-UGGUUCACCGCUCUCACUCAACAUGGCAAGGAA-3'
   |||||||||||||||||||||||||||||||||
3'-ACCAAGUGGCGAGAGUGAGUUGUACCGUUCCUU-5'

piR-98504; 29024; CDS; -136;  84; 32
5'-GCUGCUGAGGCUUCUAAGAAGCCUCGGCAAAA-3'
   |||||||||| |||||||||| ||||||||||
3'-CGACGACUCUCAAAGUUCUUUUAAGUCGUUUU-5'
spiR-98504; 29024; CDS; -162; 100; 32
5'-GCUGCUGAGGCUUCUAAGAAGCCUCGGCAAAA-3'
   ||||||||||||||||||||||||||||||||
3'-CGACGACUCCGAAGAUUCUUCGGAGCCGUUUU-5'

piR-3218674; 29475; CDS; -138; 81; 34
5'-AUGAUUUCUCCAAACAAUUGCAACAAUCCAUGAG-3'
   ||||||| ||||| |||| | ||||||||||| |
3'-UACUGAAUAGGUUAGUUACCUUUGUUAGGUACGU-5'
spiR-3218674; 29475; CDS; -170; 100; 34
5'-AUGAUUUCUCCAAACAAUUGCAACAAUCCAUGAG-3'
   ||||||||||||||||||||||||||||||||||
3'-UACUAAAGAGGUUUGUUAACGUUGUUAGGUACUU-5'
```
