# Supplementary material for: In Silico Study of piRNA Interactions with the SARS-CoV-2 Genome
Source: Int J Mol Sci. 2022 Aug 31;23(17):9919. doi: 10.3390/ijms23179919 (PMC9456458; doi:10.3390/ijms23179919)
Supplement: Supplementary file 1 [file ijms-23-09919-s001.zip › Table S1 7177+.pdf]

Table S1. The characteristics of the interaction of 70 piRNA and spiRNA BSs cluster in CDS gRNA of SARS-CoV-2 from 7175 nt to 7202 nt, 28 nt long

piRNA	Start of BS, nt	ΔG, kJ/mol	ΔG/ΔGm, %	Length, nt	ΔG*, kJ/mol
piR-134104	7176	-102	80	26	-128
piR-136315	7176	-102	80	26	-128
piR-140212	7178	-93	80	24	-116
piR-146606	7178	-100	84	24	-119
piR-151338	7179	-96	80	24	-120
piR-152752	7177	-100	81	25	-124
piR-156867	7178	-93	80	24	-116
piR-158266	7178	-98	82	24	-120
piR-161744	7178	-96	80	24	-120
piR-170126	7178	-102	80	25	-128
piR-172551	7176	-102	80	26	-128
piR-172553	7176	-106	81	26	-131
piR-174280	7177	-102	80	25	-128
piR-175524	7177	-106	85	25	-125
piR-175963	7177	-100	81	24	-124
piR-177814	7176	-104	80	26	-130
piR-178110	7178	-98	81	24	-121
piR-178851	7178	-98	81	24	-121
piR-179510	7177	-102	81	25	-126
piR-179669	7177	-102	80	25	-128
piR-179999	7178	-96	80	24	-120
piR-180563	7177	-104	83	25	-125
piR-181322	7177	-100	81	25	-124
piR-182676	7176	-104	80	26	-130
piR-183206	7177	-102	83	25	-123
piR-184425	7178	-98	81	24	-121
piR-184542	7176	-108	84	26	-129
piR-184841	7177	-104	84	25	-124
piR-185870	7178	-100	81	24	-124
piR-186021	7177	-102	80	25	-128
piR-186087	7176	-104	82	26	-127
piR-186642	7178	-96	80	24	-120
piR-187824	7177	-102	80	25	-128
piR-188562	7177	-102	81	25	-186
piR-188611	7176	-102	80	26	-128
piR-188777	7177	-104	82	25	-127
piR-189382	7177	-102	81	26	-126
piR-189757	7176	-104	82	26	-127
piR-189772	7177	-102	81	25	-126
piR-190320	7176	-104	80	26	-130
piR-190595	7176	-106	81	26	-131
piR-190864	7177	-102	80	25	-128
piR-190896	7175	-104	80	26	-130
piR-191349	7176	-108	85	25	-127
piR-191639	7176	-102	80	26	-128
piR-193077	7177	-102	80	25	-128
piR-193433	7176	-106	83	26	-128
piR-193777	7177	-104	82	25	-127
piR-193805	7177	-100	81	25	-124
piR-194123	7176	-104	80	26	-130
piR-194172	7177	-102	80	25	-128
piR-194321	7175	-104	80	27	-130
piR-194842	7176	-102	80	26	-128
piR-382458	7176	-106	81	26	-131
piR-383400	7176	-106	81	26	-131

piR-384721	7177	-98	81	25	-121
piR-390277	7178	-98	82	24	-120
piR-394136	7177	-98	81	25	-121
piR-394484	7176	-108	82	26	-132
piR-399512	7177	-98	82	24	-120
piR-402116	7176	-104	82	26	-127
piR-402789	7176	-106	81	26	-131
piR-403633	7176	-110	85	26	-130
piR-403847	7176	-106	82	26	-129
piR-403884	7176	-108	82	26	-132
piR-404198	7175	-108	82	27	-132
piR-404909	7176	-102	81	26	-126
piR-409985	7176	-106	82	26	-129
piR-410131	7176	-104	80	26	-130
piR-2498460	7178	-98	81	25	-121
Note. ΔG* is ΔG for spiRNA with ΔG/ΔGm equal to 100%.					
